# COVID-19-related anxiety in people living with HIV: an online cross-sectional study

**DOI:** 10.3906/sag-2006-140

**Published:** 2020-12-17

**Authors:** Özlem KUMAN TUNÇEL, Hüsnü PULLUKÇU, Hüseyin Aytaç ERDEM, Behice KURTARAN, Selin Ece TAŞBAKAN, Meltem IŞIKGÖZ TAŞBAKAN

**Affiliations:** 1 Department of Psychiatry, Faculty of Medicine, Ege University, İzmir Turkey; 2 Department of Clinical Microbiology and Infectious Diseases, Faculty of Medicine, Ege University, İzmir Turkey; 3 Department of Clinical Microbiology and Infectious Diseases, Faculty of Medicine, Çukurova University, Adana Turkey; 4 Faculty of Medicine, İzmir University of Economics, İzmir Turkey

**Keywords:** Anxiety, COVID-19, emotional response, HIV, pandemic, uncertainty

## Abstract

**Background/aim:**

The emergence of the coronavirus disease 2019 (COVID-19) outbreak has had an enormous emotional impact on some vulnerable groups, such as people living with human immunodeficiency virus (HIV) (PLHIV). This study was planned with the aim of assessing the anxiety levels of PLHIV and the sources of their anxiety.

**Materials and methods:**

A web-based questionnaire was sent to PLHIV using the virtual snowball sampling method. The questionnaire included questions about sociodemographic status, information about HIV infection, and the Beck Anxiety Inventory (BAI). Additionally, some opinions of the participants about COVID-19 were asked.

**Results:**

A total of 307 respondents, with a median age of 33 years, from 32 different cities, participated in the study. More than half of the respondents reported the belief that COVID-19 was not sufficiently well-known by the medical community and nearly 45% believed that they would have more complications if they contracted COVID-19. One-fourth of the participants had anxiety. Having a preexisting psychiatric disorder, perceiving that they were practicing insufficient preventive measures, not being sure about the presence of any individuals with COVID-19 in their environment, and living with a household member with a chronic disease were found to be the risk factors of PLHIV for having anxiety during this pandemic. The BAI scores were correlated with the patient-reported anxiety levels about the spread of COVID-19 in Turkey, acquiring COVID-19, transmitting COVID-19 to another person, and transmitting HIV to another person. Among the stated conditions, the most common concern was the spread of COVID-19 all over the country, while the least common was transmitting HIV to someone else.

**Conclusion:**

The results revealed that a significant proportion of the sample had anxiety, and the findings were essential for developing evidence-based strategies for decreasing the anxiety of PLHIV, especially for those who had risk factors and to provide them with better health care during this pandemic or other pandemic-like crises.

## 1. Introduction

The first case coronavirus disease 2019 (COVID-19) detected in Turkey was on 10 March 2020, and approximately 3 months prior to that, the first cases had been identified in China’s Wuhan-Hubei Province [1]. After being reported by the World Health Organization that some suspicious cases had been detected in China, very important control measures were put into place by the Turkish Government [2,3]. At the beginning of the outbreak, the frightening news on social media had caused a panic[4]. After the implementation of a successful control system over the virus, Turkey is now in the normalization process. According to official numbers, there were 17,5218 cases and 4778 deaths in Turkey, as of 12 June 2020.

The first presented patient with coinfection of severe acute respiratory syndrome coronavirus 2 (SARS-CoV-2) and human immunodeficiency virus (HIV) was diagnosed with HIV positivity after acquiring the coronavirus infection [5]. Other case reports, case series, and comments followed this publication [6–10]. Altuntaş Aydın et al. reported 4 cases of PLHIV coinfected with SARS-CoV-2 from Turkey [11]. Vizcarra et al. found a rate of coinfection in PLHIV of 1.2%–1.8%, and their results did not support the previous suggestions that PLHIV might be protected from worse outcomes [12].

The COVID-19 pandemic has had an enormous emotional impact on some vulnerable groups, such as PLHIV [13]. Some reports from China have shown that PLHIV were concerned with HIV-specific protective measures, medication shortages, and the need for psychosocial support [14,15]. The COVID-19 outbreak is expected to create extra physical and psychosocial burden for PLHIV. Taking all of these existing data into consideration, this study was planned with the aim of investigating the anxiety levels of PLHIV and define their needs during the COVID-19 pandemic.

## 2. Materials and methods

This study was performed in collaboration with the Infectious Diseases and Clinical Microbiology Department and Psychiatry Department of Ege University Faculty of Medicine.

### 2.1. Participants

The sample consisted of 307 PLHIV. The virtual snowball sampling method was used. A web-based questionnaire was first sent to patients who were being followed-up for HIV infection at the Infectious Diseases and Clinical Microbiology Department of Ege University Hospital. These patients were asked to send the questionnaire to other PLHIV who were in their social network. All responses fulfilling the inclusion criteria were analyzed. The inclusion criteria comprised 1) having a diagnosis of HIV infection, 2) being above 18 years of age, and 3) volunteering to complete the survey.

### 2.2. Procedure

This study was approved by the Ege University Research Ethics Committee (4 April 2020; 99166796-050.06.04). A Google form was designed for data collection regarding the sociodemographic status, information about HIV infection, such as the time duration since having been diagnosed, medication compliance, and knowledge level about COVID-19. Additionally, the Beck Anxiety Inventory (BAI), which is a 21-item self-report test that measures the severity of anxiety, was added [16]. In the BAI, scores can range from 0 to 63, with higher numbers suggesting greater degrees of anxiety. The suggested cut-off for clinically significant anxiety on the BAI is 16 [17], and participants who had a BAI score higher than 16 were determined as having anxiety in the current study. The Turkish version of the BAI was found to be valid and reliable [18].

Participation in this study was anonymous. Data were collected within a period of 1 week, from 6 April 2020 to 13 April 2020, to minimize the influence of rapid changes during the pandemic. Apart from the BAI, participants were requested to rate their anxiety level between 1 and 10, for the spread of COVID-19 in Turkey, acquiring coronavirus, transmitting coronavirus to another person, and transmitting HIV to another person.

### 2.3. Statistics

Data analyses were performed using IBM SPSS for Windows 21.0 (IBM Corp., Armonk, NY, USA). The Kolmogorov–Smirnov test was used to test normality. All of the quantitative variables had skewed distribution; therefore, they were expressed as the median and minimum–maximum values. Categorical variables were presented as frequency and percentage. The Mann–Whitney U or Kruskal–Wallis tests were used to compare the quantitative data. For comparison of the categorical variables, the χ² test or Fisher exact test was used. The Spearman rank test was used to assess correlations between continuous variables. P < 0.05 was considered statistically significant. For the multivariate analysis, a backward stepwise logistic regression procedure was performed. The Chronbach alpha test was used to measure the internal consistency reliability of the BAI in the studied sample.

## 3. Results

### 3.1. Sociodemographic variables

Among the 307 respondents, 289 (94.1%) were male. The median age was 33 (between 18 and 77) years. Participants from 32 different cities answered the questionnaire, 203 (66.1%) of whom lived in crowded cities with a population of over 1 million. Of the participants, 97 (31.6%) lived alone, 98 (31.9%) lived with their families, 35 (11.4%) lived with a person aged 65 years or older. Additionally, 24 (7.8%) were students, 23 (7.5%) were unemployed, 14 (4.6%) were retired, and the remainder were employed. Among the employed participants, 20 were health care workers.

### 3.2. Answers related to HIV and other medical-psychiatric conditions

The time since diagnosis of HIV infection was less than 1 year in 64 (20.8%) of the participants, 1–5 years in 194 (63.2%) of the participants, 5–10 years in 33 (10.7%) of the participants, and longer than 10 years in 16 (5.2%) of the participants. Moreover, 304 (99%) of the participants were taking their medication for HIV properly.

Of the respondents, 71 (23.1%) stated that they had another physical disease, and 42 (13.7%) had a psychiatric disorder. Additionally, 101 (32.9%) participants had at least 1 household member with a chronic disease.

### 3.3. Answers related to COVID-19

Of the participants, 139 (45.3%) reported that proper precautions were taken to prevent the spread of COVID-19 in their workplace. Other answers of the participants to the questions about COVID-19 are shown in the Table 1. The participants were asked about 13 selected precautions which were endorsed by the Turkish Ministry of Health, to prevent COVID-19 transmission (Table 2). The median number of followed recommendations was 10 (between 0 and 13).

**Table 1:** Answers of the participants to the question related to COVID-19.

Questions	Yes		No		No idea/Do not know	
	n	%	n	%	n	%
Are there any individuals with COVID-19 around you?	19	6.2	183	59.6	105	34.2
Do you think COVID-19 is sufficiently well-known by the medical community?	106	34.5	169	55	32	10.4
Do you think you will have more complications if you acquire COVID-19, as you are HIV+?	130	42.3	132	43	45	14.7
Have you been trained by your employer on how to protect yourself against COVID-19?	82	47.1	92	30	-	-
Is your personal protective equipment sufficient to protect you against COVID-19?	234	76.2	73	23.8	-	-
Do you think you have taken enough precautions to protect yourself from COVID-19?	248	80.8	59	19.2	-	-

**Table 2:** Precautions that are taken to prevent the transmission of COVID-19.

What kind of precautions do you take to prevent COVID-19 transmission?	n	%
I wash my hands with soap often, at least for 20 s.	296	96.4
I avoid close contact, such as hugging and shaking hands with people.	277	90.2
I do not go out unless it is necessary.	260	84.7
I always cover my mouth and nose with a disposable tissue when I cough or sneeze. If a tissue is not accessible, I cover my mouth and nose with the inside of my elbow.	252	82.1
I keep a distance of 3–4 m from others who have flu-like symptoms.	235	76.5
I avoid touching my eyes, nose, and mouth with unwashed hands.	225	73.3
I drink lots of water, eat a balanced diet, and pay attention to my sleep patterns.	222	72.3
I do not share my personal belongings, such as a towel.	219	71.3
I ventilate my indoor environment, frequently.	218	71
I eat a variety of foods and use medication to boost my immune system.	184	59.9
I clean frequently touched surfaces such as doorknobs, light switches, countertops, and handles with water and detergent, every day.	170	55.4
I wash my clothes at 60–90°C.	165	53.7
I do not go out without wearing a mask.	128	41.7

### 3.4. Results related to the anxiety scores

Participants were required to rate their anxiety levels for some of the stated conditions. The scores of these questions are shown in Table 3.

**Table 3:** Anxiety levels of the participants for some stated conditions.

Sources of anxiety	Minimum	Median	Maximum
The spread of COVID-19 in Turkey	1	6	10
Acquiring coronavirus	1	4	10
Transmitting coronavirus to another person, because of having undiagnosed COVID-19	1	5	10
Transmitting HIV to another person	1	3	10

Participants were required to rate their anxiety level between 1 and 10 for these questions, where 1 represents the lowest, and 10 represents the highest level of anxiety.

For the BAI, the Cronbach alpha was calculated as 0.939. The median BAI score was 7 (between 0 and 62) for the whole sample. Additionally, 79 (25.7%) of the participants were defined as having anxiety, as the result of having a BAI score higher than 16.

There was no correlation between age and anxiety level. On the other hand, all of the anxiety scores that were gained from either the BAI or the questions shown in Table 3 were intercorrelated (Table 4). The percentage of followed precautions among the 13 selected measures was inversely correlated with the BAI score (P = 0.006, r = –0.156). There was no correlation between this percentage and the other patient-reported anxiety levels.

**Table 4:** Correlations between the anxiety scores.

Source of anxiety	Age	Spread in Turkey	Acquiring COVID-19	Transmitting COVID-19	Transmitting HIV
Spread in Turkey	P =0.357 r = 0.053	r = 1			
Acquiring COVID-19	P = 0.054 r = 0.110	P < 0.0001 r = 0.778	r = 1		
Transmitting COVID-19	P = 0.320 r = 0.057	P < 0.0001 r = 0.790	P < 0.0001 r = 0.711	r = 1	
Transmitting HIV	P = 0.290 r = 0.061	P < 0.0001 r = 0.384	P < 0.0001 r = 0.339	P < 0.0001 r = 0.521	r = 1
BAI	P = 0.402 r = –0.048	P < 0.0003 r =0.205	P < 0.0001 r =0.270	P = 0.001 r =0.193	P = 0.006 r = 0.157

There were no statistically significant relationships between the BAI scores and gender, living with a household member older than 65 years of age, time since HIV diagnosis, or having another chronic disease. The variables that had significant associations with the BAI score are shown in Table 5.

**Table 5:** Answers that had significant associations with the Beck Anxiety Inventory scores.

Questions	Answers	BAI score		Statistics
		Med	Min–Max	
Do you have a psychiatric disorder?	Yes	14.5	0–51	P < 0.0001Z = –4.292
	No	6	0–62	
Is there anyone else with a chronic disorder in your house?	Yes	9	0–51	P = 0.005 Z = –2.814
	No	6	0–62	
Are there any individuals with COVID-19 around you?	a) Yes	13	0–39	P = 0.011 a-b: P = 0.128, Z = –1.524 a-c: P = 0.690, Z = –0.399 b-c: P = 0.005, Z = –2.818
	b) No	6	0–62	
	c) Do not know	9	0–44	
Do you think COVID-19 is sufficiently well-known by the medical community?	a) Yes	6	0–62	P = 0.034 a-b: P = 0.016, Z = –2.407 a-c: P = 0.923, Z = –0.096 b-c: P = 0.124, Z = –1.540
	b) No	8	0–51	
	c) No idea	4.5	0–51	
Have you been trained by your employer on how to protect yourself against COVID-19?	Yes	5	0–50	P = 0.009 Z = –2.613
	No	8	0–42	
Do you think you will have more complications if you acquire COVID-19, as you are HIV+?	a ) Yes	9	0–62	P = 0.002 a-b: P < 0.0005, Z = –3.511 a-c: P = 0.224, Z = –1.215 b-c: P = 0.218, Z = –1.232
	b) No	6	0–47	
	c) Do not know	6	0–51	
Is your personal protective equipment sufficient to protect you against COVID-19?	Yes	7	0–51	P = 0.038Z = –2.079
	No	8	0–62	
Do you think you have taken enough precautions to protect yourself from COVID-19?	Yes	6	0–62	P = 0.001 Z = –3.253
	No	12	0–50	

BAI: Beck Anxiety Inventory score, med: median, min: minimum, max: maximum

The characteristics of the participants who had anxiety during the COVID-19 pandemic were analyzed. There were no statistically significant differences between the participants who had anxiety and the rest of the sample with regards to gender, living with a household member older than 65 years of age, duration since receiving HIV diagnosis, having another chronic disease, and being trained by their employer about protection against COVID-19. The variables that had a significant relationship with having anxiety are given in Table 6.

**Table 6:** Answers that had a significant relationship with having anxiety.

Questions and Answers		No anxiety		Anxiety		Statistics
		n	%	n	%	
Do you have a psychiatric disorder?	Yes	22	52.4	20	47.6	P = 0.001, χ² = 10.905
	No	206	77.7	59	22.3	
Is there anyone else with a chronic disorder in your house?	Yes	67	66.3	34	33.7	P = 0.026, χ² = 4.953
	No	161	78.2	45	21.8	
Are there any individuals with COVID-19 around you?	a) Yes	11	57.9	8	42.1	P = 0.018, χ² = 8.005 a-b: P = 0.041 *a-c: P = 0.575, χ² =0.314 b-c: P = 0.021, χ² = 5.313
	b) No	146	79.8	37	20.2	
	c) Do not know	71	67.6	34	32.4	
Do you think COVID-19 is sufficiently well-known by the medical community?	a) Yes	88	83	18	17	P = 0.037, χ² =6.587 a-b: P = 0.011, χ² = 6.257 a-c: P = 0.255,χ² = 1.940 b-c: P = 0.929,χ² = 0.008
	b) No	117	69.2	52	30.8	
	c) No idea	23	71.9	9	28.1	
Do you think you will have more complications if you acquire COVID-19, as you are HIV+?	a) Yes	87	66.9	43	33.1	P = 0.013, χ² = 8.672 a-b: P = 0.004,χ² = 8.515 a-c: P = 0.739,χ² = 0.111 b-c: P = 0.151,χ² = 2.061
	b) No	109	82.6	23	17.4	
	c)Do not know	32	71.1	13	28.9	
Is your personal protective equipment sufficient to protect you against COVID-19?	Yes	181	77.4	53	22.6	P = 0.039, χ² = 4.240
	No	47	64.4	26	35.6	
Do you think you have taken enough precautions to protect yourself from COVID-19?	Yes	195	78.6	53	21.4	P = 0.001, χ² = 11.687
	No	33	55.9	26	44.1	

*: Fisher exact test (2-sided) was applied.

A backward stepwise logistic regression procedure was performed to gauge the combined impact of the categorical variables on having anxiety. As a result, having anxiety was found to be significantly associated with having a psychiatric disorder (P = 0.002, OR = 3.02, 95% confidence interval (CI) = 1.49–6.13), the perception of taking insufficient precautions to protect oneself from COVID-19 (P = 0.002, OR = 2.75, 95% CI = 1.47–5.16), being unsure about the presence of an individual with COVID-19 near oneself (P = 0.023, OR = 1.94, 95% CI = 1.09–3.44), and living with a household member with a chronic disease (P = 0.022, OR = 1.92, 95% CI = 1.10–3.37).

## 4. Discussion

In this challenging time, it was determined that PLHIV, who had a preexisting psychiatric disorder, perceived that they were practicing insufficient preventive measures, were not sure about the presence of any individuals with COVID-19 in their environment, and lived with a household member with a chronic disease, were vulnerable to anxiety.

A total of 307 participants from 32 cities, with a median age of 33 years, participated in the study. Nearly 95% of the participants were male. According to the statistical records of the Turkish Ministry of Health, there were 24,209 confirmed HIV+ cases reported from 1985 to June 2019 in Turkey, and nearly 80% of these individuals were male Türkiye Cumhuriyeti Sağlık Bakanlığı Halk Sağlığı Genel Müdürlüğü Bulaşıcı Hastalıklar Dairesi Başkanlığı HIV-AIDS İstatistik [online]. Website u29db [accessed 19 July 2020].. Therefore, it can be said that the sample herein represented a substantial portion of the PLHIV in Turkey. The percentage of males in this study was slightly higher than the percentage in the general HIV population.

A study finding optimal adherence in 85% of participants revealed that antiretroviral treatment (ART) adherence by PLHIV was relatively higher in Turkey than that in other countries [19]. In the current study, much higher treatment adherence was reported. Online surveys can reduce the social desirability bias caused by the expectations of an interviewer [20]. Hence, less distortion was expected than in traditional pen and pencil surveys, as the current survey was online and anonymous [21]. This high adherence can be explained as increased engagement in healthy behavior, which is promoted by a high-risk perception related to COVID-19, using the health belief model [22]. Moreover, anti-HIV drugs have been suggested to be effective against SARS-CoV-2 [6,7], and this information may also be understood by the PLHIV, yielding to improvement in their compliance. Additionally, the internet-savvy sample herein may have been highly educated about HIV and ART, which is an important factor for treatment adherence [23]. Regardless, it should always be kept in mind that patient-reported treatment adherence ratios may be somewhat distorted.

When the answers of the participants to the questions about COVID-19 were analyzed, it was found that nearly half of the participating PLHIV believed that they were in the risk group for COVID-19. Likewise, some researchers have claimed that PLHIV were vulnerable to COVID-19 [5,24–26], whereas others have suggested that treatment-adherent PLHIV may have a lower risk than the general population [8,9,27]. Although more than half of the participants believed that COVID-19 was not sufficiently well-known by the medical community, more than 75% of the participants assumed that they had sufficient personal protective equipment and had taken enough precautions to protect themselves from COVID-19. These answers can be interpreted as a psychological adjustment to a pandemic. Although the participants evaluated themselves as vulnerable and appraised the pandemic as ambiguous, they use problem focused-coping [22]. Taking some actions and following the recommendations may have given them a sense of control over the virus. These answers seemed to have been related to coping responses that have also been seen in previous outbreaks [28].

Nearly half of the participants followed all of the selected recommendations by the Turkish Ministry of Health. The most followed recommendation was about hand washing, while the least adopted recommendation was wearing a mask. This result may have been attributed to the changing recommendations of the Turkish Ministry of Health during the course of the pandemic, wherein initially only individuals who had symptoms of COVID-19 were advised to wear masks, followed later by a recommendation for everybody to do so.

Participants were required to rate their anxiety level for some statements about COVID-19 and HIV. The most common concern for the participants was the spread of the virus all over the country. This concern may have been related to the possibility of experiencing some barriers to engagement, along the HIV care continuum as the result of increased demand and pressure on health care services. Although there have been no problems in terms of the supply of medication in Turkey, in many countries, PLHIV are at risk of the discontinuation of ART [14, 29]. Moreover, preliminary results of a study from Florida showed that older PLHIV were worried about the impact of COVID-19 on their health [30]. It is noteworthy that the score for concern about acquiring COVID-19 was less than that for transmitting it to someone else unintentionally. This finding may have been the result of the selfstigmatization related to HIV, which is characterized by feelings of guilt and shame [31]. The most striking result was the ranking of the anxiety level about transmitting HIV to another person, which was placed at the bottom of the list. The PLHIV had more anxiety about transmitting SARS-CoV-2 to someone else than they had about transmitting HIV. At first glance, this result may seem strange. However, it is known that uncertainties bring on anxiety and SARS-CoV-2 is a new virus with many unknowns [32]. On the other hand, PLHIV are aware of ways to prevent HIV transmission. The concept of ‘undetectable equals untransmittable’ encourages engagement in care, and PLHIV in Turkey have been mostly educated about preexposure and postexposure prophylaxis, which prevents the spread of HIV [23,33]. Hence, the median anxiety level for transmitting HIV to someone scored only 3 out of 10 in this highly treatment-adherent sample.

The outcomes of the correlative analysis indicated that all of the anxiety scores were intercorrelated. From these intercorrelations, it can be inferred that COVID-19 is one of the main concerns of the participants. Furthermore, it was found that the percentage of followed precautions was inversely correlated with the BAI score, which showed that engaging in specific preventative health actions was inversely correlated with the level of anxiety. Consistent with these results, Taha et al. found that problem-focused coping was negatively related to H1N1-related anxiety in the general population [22].

Associations between the sociodemographic and clinical characteristics of the sample and developing anxiety in PLHIV during COVID-19 pandemic were analyzed. When the outcomes of the questions with 3 choices were examined, it is obtained that the PLHIV who believed that COVID-19 was not sufficiently well-known by the medical community had greater anxiety than those who believed the opposite to be true. This result was not surprising, as it is known that when the nature of a threat is not well understood, it is a source of distress [22]. Similarly, during the SARS outbreak, studies involving infected nurses and the parents of infected children showed that uncertainty regarding outcomes, side effects, and efficacy were related to increased anxiety [34,35]. Likewise, answering the question “Are there any individuals with COVID-19 around you?” as “Do not know” yielded greater anxiety scores than the answer “No”. Being unsure about this situation led to greater anxiety. PLHIV who perceived themselves as vulnerable to COVID-19 had greater anxiety than participants who believed that they were not in the risk group. This result was consistent with the findings of chronically ill patients during the SARS outbreak [36].

The prevalence of anxiety and/or fear was found to be 3.2%–12.6% in the SARS, H1N1, and Ebola-related investigations in different populations [28]. However, in the current study, one-fourth of the participants had a score higher than 16, which meant that they had anxiety. This prevalence, which was much higher than that found during previous outbreaks, showed the importance of implementing new interventions that are specialized for PLHIV, to decrease their anxiety.

It was attempted herein to define the risk factors of having anxiety for PLHIV at the time of the COVID-19 pandemic. The greatest risk was for those with psychiatric disorders, which was an expected finding, as health-related threats or physical distancing can be particularly challenging for people with preexisting psychiatric disorders Center for the Study of Traumatic Stress CSTS (2020) Taking care of patients during the coronavirus outbreak: A guide for psychiatrists [online]. Website u29dd[accessed 30 April 2020]. Moreover, PLHIV who perceived themselves as taking insufficient precautions, did not know exactly if there was an individual with COVID-19 around them, and lived with a household member who had a chronic disease had greater probability of having anxiety. People who cannot take sufficient precautions may have anxiety; on the other hand, people with anxiety may underestimate the actions they have taken. Similarly, people living in a place where the number of confirmed cases is high, may be uncertain about the presence of an individual with COVID-19 near them, and therefore, may have more anxiety, and people that have anxiety may not be sure about a condition like this. As it is known that people with chronic diseases are more vulnerable to COVID-19, it is understandable that PLHIV who live with a loved one who has a chronic disease would have higher anxiety.

This study had some limitations. First of all, there were biases associated with the data collection method, such as sampling error and response bias. Selection bias related to the internet-savvy population also decreased the generalizability of the results. The study also had self-reporting bias. Face-to-face interviews might have decreased answer falsification, whereas during an outbreak, online surveys are safer for the participants and provide the chance to access to a large sample of a specific stigmatized population, such as PLHIV. As the data were collected anonymously, their clinical records could not be checked. The striking male predominance led to another limitation. Furthermore, since the study was cross-sectional, it was not possible to detect a certain causal relationship between some characteristics and anxiety for PLHIV during the COVID-19 pandemic. Having no control group also made it difficult to distinguish HIV-specific concerns from worries generalized in the whole population. On the other hand, the results of the study were important for developing evidence-based strategies to provide better health care to PLHIV during this pandemic or other pandemic-like crises. Understanding their concerns will aid in the implementation of feasible interventions.

In conclusion a significant percentage of the sample comprising PLHIV had anxiety. Participants who had a preexisting psychiatric disorder, the perception of taking insufficient precautions to protect oneself, uncertainty about the presence of an individual with COVID-19 around oneself, and lived with a household member who had a chronic disease were at greater risk of experiencing anxiety during this pandemic.
